# Integration of Nodal and BMP Signals in the Heart Requires FoxH1 to Create Left–Right Differences in Cell Migration Rates That Direct Cardiac Asymmetry

**DOI:** 10.1371/journal.pgen.1003109

**Published:** 2013-01-24

**Authors:** Kari F. Lenhart, Nathalia G. Holtzman, Jessica R. Williams, Rebecca D. Burdine

**Affiliations:** 1 Department of Molecular Biology, Princeton University, Princeton, New Jersey, United States of America; 2 Department of Biology, Queens College, City University of New York, Flushing, New York, United States of America; University of Pennsylvania School of Medicine, United States of America

## Abstract

Failure to properly establish the left–right (L/R) axis is a major cause of congenital heart defects in humans, but how L/R patterning of the embryo leads to asymmetric cardiac morphogenesis is still unclear. We find that asymmetric Nodal signaling on the left and Bmp signaling act in parallel to establish zebrafish cardiac laterality by modulating cell migration velocities across the L/R axis. Moreover, we demonstrate that Nodal plays the crucial role in generating asymmetry in the heart and that Bmp signaling via Bmp4 is dispensable in the presence of asymmetric Nodal signaling. In addition, we identify a previously unappreciated role for the Nodal-transcription factor FoxH1 in mediating cell responsiveness to Bmp, further linking the control of these two pathways in the heart. The interplay between these TGFβ pathways is complex, with Nodal signaling potentially acting to limit the response to Bmp pathway activation and the dosage of Bmp signals being critical to limit migration rates. These findings have implications for understanding the complex genetic interactions that lead to congenital heart disease in humans.

## Author Summary

Defects in left–right (L/R) patterning can lead to severe defects in the formation of the heart. In fact, three of the most common forms of congenital heart disease, transposition of the great arteries, chamber septation defects, and chamber isomerisms, can be caused by earlier defects in L/R asymmetry. The Nodal and Bmp signaling pathways influence the development of cardiac asymmetry, but how these signals function in this process is not well understood. In this report, we have clarified the specific roles for the Nodal versus Bmp pathways in the heart. We find that Nodal signals increase the rate of cardiac cell migration, while Bmp signals decrease cardiac cell velocities. We demonstrate that asymmetric Nodal signaling plays a critical role in directing asymmetry in the heart in contrast to reports suggesting that signaling via Bmp4 is the more critical pathway. In fact, we find that Bmp4 signaling is dispensable for correct asymmetry in the heart in the presence of asymmetric Nodal signals. In addition, we have identified a novel integration between these two pathways at the level of the transcription factor FoxH1, which is required for cardiac cell responsiveness to both Nodal and Bmp signals. Taken together, this work significantly increases our understanding of how the signals regulating cardiac asymmetry function and integrate to consistently establish cardiac laterality. These results also suggest that human congenital heart defects that have not been found to result from single mutations within individual genes may develop due to combinations of mutations within components of these two separate pathways.

## Introduction

Establishment of asymmetries along the left–right (L/R) axis is critically important for proper placement, morphogenesis and functioning of vertebrate organs [1], [2]. In the heart, defects in early L/R patterning events are implicated in three of the six most common forms of congenital heart disease (CHD): transposition of the great arteries, chamber septation defects and chamber isomerisms [3], [4], [5]. Among the genetic lesions known to associate with cardiac defects are mutations in a number of proteins within the Nodal signaling pathway [6]; the asymmetric activation of which plays a conserved role in specifying the L/R axis in all vertebrates [1], [2]. Human mutations in Bmp pathway genes have also been implicated in the development of CHD [7].

The zebrafish heart develops from cardiac precursors derived from the lateral plate mesoderm (LPM) which migrate to the midline and fuse to form the cardiac cone [8]. Active cell migration within the cardiac cone converts this symmetric, disc-shaped structure into the asymmetric, linear heart tube, with these cellular movements being regulated by Nodal and Bmp signals [9], [10], [11], [12]. However, significant controversies exist concerning the relative requirements for these two pathways. While the laterality of Nodal signaling has been shown to influence the direction of cardiac cell migration [9], the Bmp pathway has been implicated as providing the dominant laterality cue to the heart [12]. Therefore, a complete understanding of asymmetric cardiac morphogenesis requires clarification of the specific requirements for and interactions between these two TGFβ signaling pathways.

Here, we report the identification of separate and parallel functions for the Nodal and Bmp pathways in establishing consistent cardiac asymmetry. We find that the Nodal signaling pathway provides the dominant laterality cue to the heart and directs cardiac cell migration by increasing cell velocities on the left of the wild type (WT) cardiac cone. By contrast, we find the Bmp pathway negatively regulates cardiac cell migration rates. The resulting asymmetries in cell velocities across the L/R axis result in the rotation of the cardiac cone to produce the asymmetrically jogged heart. Our findings are particularly important in clarifying the requirements for Bmp signaling in establishing cardiac laterality. Our results are consistent with a role for BMP signaling in limiting cell migration on the right side of the cardiac cone, while the Bmp pathway has historically been thought to positively regulate cell migration and act predominantly on the left of the developing heart [12], [13], [14]. We also demonstrate that Bmp4 is dispensable for establishment of cardiac asymmetry in the presence of asymmetric Nodal signaling, suggesting that Nodal plays the more critical role in this process. However, in the absence of Nodal signaling, the heart is extremely sensitive to the dosage of Bmp4 signals, a finding with significant implications for the existence of combinatorial mutations in multiple pathways giving rise to CHD in humans. Finally, we have identified the existence of a novel integration between these two TGFβ pathways. Through genetic analysis, we find that the “Nodal” transcription factor FoxH1 is required for both Nodal-dependent and independent functions within the heart and that, separate from its requirement to transduce Nodal signals, FoxH1 is also necessary to mediate Bmp responsiveness within cardiac cells. Taken together, this work greatly enhances our understanding of the specific requirements for Nodal versus Bmp signaling in establishing cardiac asymmetry and provides new insight into the cross-regulation and integration between these two pathways necessary for the consistent development of proper cardiac laterality.

## Results/Discussion

### Nodal signaling establishes cardiac L/R asymmetry by increasing myocardial migration rates on the left

**Figure 1:**
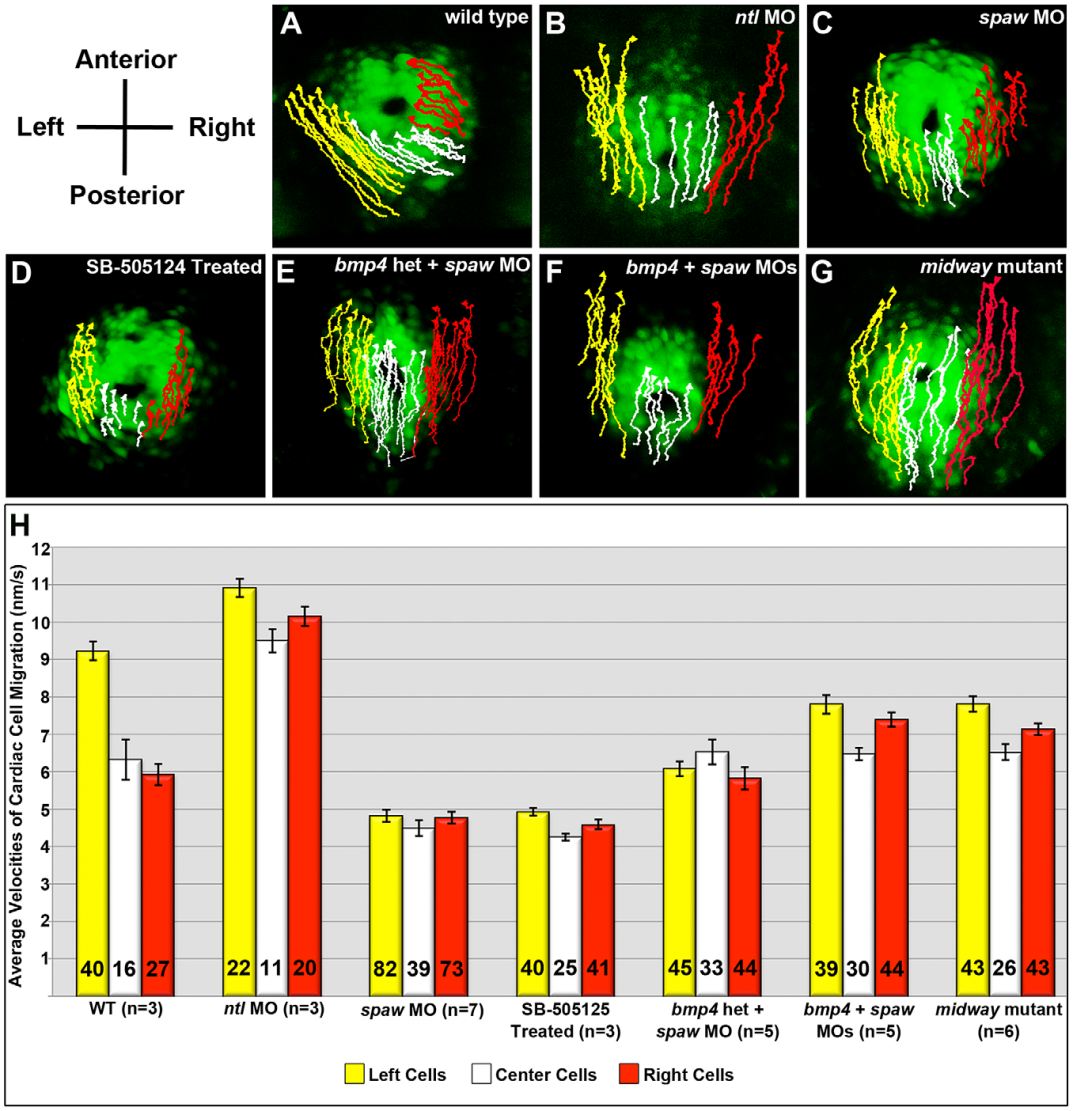
Trajectories and average velocities of migrating cells within the cardiac cone. A–G: First frame of time lapse movies taken from a dorsal view of *Tg(myl7:eGFP)* embryos overlaid with cell trajectories. Yellow tracks: left cells. White tracks: center cells. Red tracks: right cells. A: All cells exhibit trajectories directed toward the anterior and left in WT embryos. B: Bilateral expression of *spaw* in *ntl* morphants leads to loss of L/R asymmetry in cardiac cell migrations. C–G: L/R directionality is also absent in embryos C: lacking the Nodal ligand *spaw*; D: treated with the SB-505124 Nodal inhibitor drug E: lacking *spaw* and one functional copy of *bmp4*; F: injected with both *bmp4* and *spaw* morpholinos; and G: homozygous *midway* mutants. H: Numbers within each bar indicate the number of cells tracked on the left, center and right for all embryos of each genotype. The number of embryos utilized in analysis is indicated (n = ) for each genotype. WT embryos exhibit differences in average cell velocities along the L/R axis, with left cells migrating significantly faster than right cells. Bilateral exposure to Spaw leads to significant increases in average velocities in left and right cells of *ntl* morphants, while loss of Spaw and global inhibition of Nodal signaling through drug treatment results in significantly decreased rates of migration. In embryos with diminished Bmp signaling along with loss of Spaw, average cell velocities are significantly increased compared with loss of Spaw alone. Error bars in H indicate standard error of the mean. MO: morpholino. Het: Heterozygotes.

**Figure 2:**
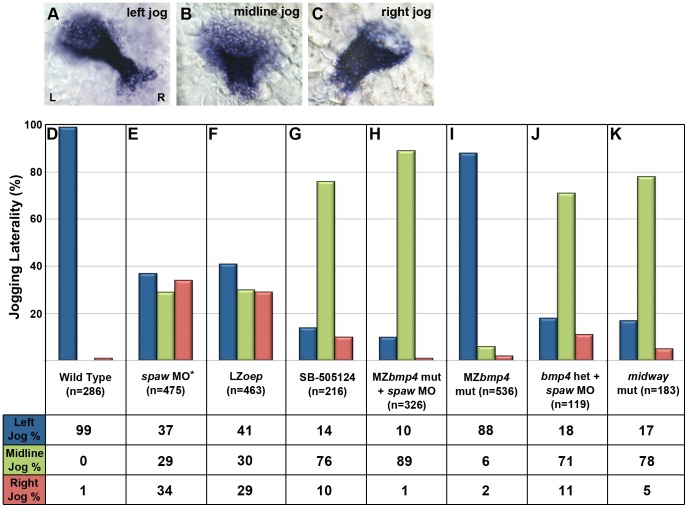
Jogging laterality phenotypes. Embryos were scored for laterality of cardiac jogging between 24 and 30 hpf. A–C: Dorsal views of the heart at 24 hpf showing jogging laterality phenotypes by RNA in situ hybridizations using the *myl7* probe D: WT embryos exhibit primarily left-directed cardiac jog, as do *bmp4^Y180^* mutants (I) lacking both maternal and zygotic *bmp4* (MZ*bmp4*). E,F: While WT embryos injected with *spaw* morpholino [9] and late-zygotic (LZ) *oep* mutants display randomized jogging laterality, H: MZ*bmp4^Y180^* mutants injected with *spaw* morpholino lack significant jogging asymmetries. I: Loss of *bmp4* alone does not affect jogging laterality and resembles WT (D) suggesting Bmp is dispensable for correct jogging when asymmetric Nodal signals are present. J: Jogging laterality in the absence of Spaw is highly sensitive to the level of Bmp4 present, as loss of a single functional copy of *bmp4* in embryos deficient for Spaw is sufficient to result in predominantly midline jogging phenotypes. K: *midway* mutants homozygous for a nonsense mutation in the Nodal transcription factor FoxH1 display jogging phenotypes similar to what is observed in embryos lacking both Nodal and Bmp signaling. We note that a discrepancy exists between the randomized jogging of *spaw* morphants and the predominantly midline jogging of SB-505124-treated embryos (G). As described in more detail in the discussion, these phenotypic differences are likely due to the more global inhibition of TGFβ signaling achieved through drug treatment and we believe reflects the involvement of a second, as yet unidentified, TGFβ ligand in establishing jogging laterality. MO: morpholino. Het: Heterozygotes. Mut: Mutant. Asterisk: jogging data presented supplementary to [9].

We have previously shown that the sidedness of Nodal signaling directs the first asymmetry evident in the heart; a left, anterior-directed movement of atrial cells within the zebrafish “cardiac cone” [9], also see Figure 1A). To gain insight into how Nodal signaling establishes the laterality of cell trajectories, we analyzed cell behaviors in time lapse images of cardiac development during conversion of the cardiac cone into the linear heart tube, and find that the left-biased asymmetry of cell movements in WT embryos is established by differences in cell migration velocities along the L/R axis. Cells on the left of the cardiac cone, which in WT embryos are exposed to the zebrafish Nodal *southpaw* (*spaw*), migrate with an average rate of 9.2 nm/s (Figure 1A and 1H). Cells on the right side of the cone that do not exhibit Nodal target gene expression in WT embryos migrate with significantly slower rates of 5.9 nm/s (p<10^−3^; Figure 1H; Video S1). Thus, exposure to Nodal signaling appears to induce increases in cell velocities within the cardiac cone. This L/R asymmetry in migration rates consistently leads to a left direction of cardiac jog by 24 hours post-fertilization (hpf) in WT embryos (3/3 embryos; Figure 2A and 2D).

To confirm that Nodal signaling is responsible for the increase in migration rates in cells on the left of the WT cardiac cone, we analyzed *no tail* (*ntl*) morpholino-injected embryos (morphants), which express *spaw* bilaterally in the lateral plate mesoderm [15]. We find that *ntl* morphants exhibit a statistically significant increase in cell velocities compared with WT, with the left and right cells migrating an average of 10.9 nm/s and 10.2 nm/s, respectively (p<10^−3^; Figure 1B and 1H; Video S2). This bilateral increase in migration rates leads to a loss of asymmetry in cell trajectories and subsequent loss of asymmetry in heart position at 24 hpf (3/3 embryos analyzed) (Figure 1B; and data not shown). We have further confirmed that this velocity increase is Nodal-dependent by analyzing migration rates in *spaw* morphants and embryos treated with a Nodal-inhibitor drug, SB-505124, that blocks activity of the Nodal Type I receptors [16], [17]. In both conditions, migration rates are significantly reduced compared to WT (p<10^−3^ for each), but with no statistical difference in average cell velocities between *spaw* morphant and drug-treated embryos (p = 0.713) (Figure 1C, 1D and 1H; Video S3). These migration rates are also consistent with what has been reported for late zygotic *one-eyed pinhead* mutants (LZ*oep*), which lack the essential Nodal co-receptor [10], [18]. Importantly, the loss of biased asymmetry in migration rates in *spaw* morphants, LZ*oep* mutants, and drug-treated embryos also results in predominant loss of biased asymmetry in cardiac jogging (see below) (Figure 2E, 2F and 2G), indicating that differences in cell velocity along the L/R axis of the cardiac cone are required for consistent establishment of asymmetry in cardiac jog.

### The Bmp pathway directs random jogging asymmetry in the absence of Nodal signaling

While asymmetric *spaw* is required to establish differences in cardiac velocities along the L/R axis, the heart can respond to additional laterality cues in the absence of Nodal signaling. Loss of either Spaw or the essential Nodal co-receptor Oep leads to randomized jogging (left, right or midline), not midline jogging, suggesting residual, randomized L/R signals function in the absence of Nodal pathway activation (Figure 2A–2C, 2E and 2F) [9]. Evidence from the literature suggests that the Bmp pathway may provide this additional signal, as ubiquitous over-expression of Bmp ligands or global inhibition of the Bmp pathway both lead to alterations in cardiac laterality [12], [13], [14], [19]. If Bmp signals do provide the remaining asymmetric information in the absence of Nodal signaling, we hypothesized that combined inhibition of both the Nodal and Bmp pathways would remove all asymmetric information to the heart and lead to predominantly midline jogging. Bmp4 has been implicated as the Bmp ligand required during cardiac laterality determination [13], [14], [19], so we utilized the *bmp4^Y180^* null mutant [20] to analyze the jogging phenotypes of embryos with diminished Nodal and Bmp pathway activities.

Consistent with our hypothesis, loss of both Spaw and Bmp4 produces predominantly midline hearts by 24 hpf, with 89% of embryos lacking L/R asymmetry in cardiac jog (midline jog; Figure 2H). Previous work has suggested that Bmp signals provide the dominant laterality cue to the heart and that Nodal signaling plays a secondary role, required only to ensure a consistent bias in Bmp pathway activity [12], [14]. However, in contrast to this existing view, we find that maternal-zygotic (MZ) MZ*bmp4^Y180^* mutants on their own do not exhibit significant jogging defects (Figure 2I). This result suggests that Bmp4 is only required to provide asymmetric cues in the absence of Nodal and is otherwise dispensable for cardiac laterality. Additionally, these data argue that Spaw, not Bmp4, provides the dominant laterality cue to the developing heart. The differences in our results from those previously published are possibly because those studies utilized overexpression of Bmp2b, producing non-physiological levels of Bmp signaling via a ligand that is not expressed in the cardiac field at this stage of development. Interestingly, we find that in the absence of Spaw, cardiac cells are highly sensitive to even small changes in the dosage of *bmp4*, further supporting the idea that the levels of Bmp signaling can influence this process. While embryos containing only one copy of the *bmp4^Y180^* mutation do not exhibit jogging defects (data not shown), when Nodal signaling is inhibited in these embryos, 76% display midline hearts at 24 hpf (Figure 2J). Taken together, our results provide strong evidence that Nodal signals dominantly influence cardiac laterality. Importantly, we find Bmp4 to only be critical in the absence of Spaw to direct jogging, at which point cardiac asymmetry is highly sensitive to the overall level of Bmp signaling present in the embryo.

Our data suggests that the hearts that jog directionally in Spaw morphants (left and right; Figure 2E) are responding to asymmetric information provided by Bmp4. We predict this should result in asymmetries in cell migration velocities which produce the resulting directional jog. However, despite their randomized jogging phenotype, asymmetries in cell velocities across the L/R axis were not detected, and both the global population of *spaw* morphants and individual morphant embryos display bilaterally reduced cell velocities. Due to the profoundly diminished migration rates in *spaw* morphants, there is a subsequent delay in the conversion of the cardiac cone into the linear heart tube. Thus, it is likely that our time lapse movies were simply not long enough to observe an establishment of L/R asymmetry in cell velocities in the subset of *spaw* morphants with directional cardiac jog (3/7 embryos). In addition, even when directional jog is established in these morphants, the heart tubes are displaced from the midline much less significantly than are left-jogged hearts in WT embryos. Therefore, even when asymmetries are established in cell velocities along the L/R axis, we would anticipate those differences to be substantially less than the asymmetry observed in WT cardiac migration rates and, therefore, potentially below the threshold for significance used in our statistical analysis.

### Bmp signaling negatively influences cardiac migration rates

To determine how Bmp signaling influences cell migration during jogging, we analyzed cardiac cell migration in *bmp4^Y180−/+^* embryos injected with *spaw* MO. We find that combined loss of Nodal signaling and just one functional copy of *bmp4* leads to significantly increased average migration rates (6.08 nm/s on left and 5.82 nm/s on right) compared with loss of Nodal signaling alone (4.8 nm/s left and right; p<10^−3^; Figure 1E and 1H; Video S4). This increase in cardiac migration rate is even more pronounced in embryos injected with *bmp4* and *spaw* MOs, with left- and right-sided cells displaying average velocities of 7.8 nm/s and 7.4 nm/s, respectively (Figure 1F and 1H; Video S5). As with all other conditions in which significant L/R asymmetries in migration rate are lost, nearly all embryos with combined inhibition of *bmp4* and *spaw* also exhibit loss of directional cardiac jog (midline jog −3/5 *bmp4^Y180−/+^* embryos injected with *spaw* MO; 4/5 *bmp4/spaw* double morphants). The increase in cardiac migration velocities upon inhibition of the Bmp pathway suggests that Bmp signaling is normally required to limit the migratory ability of cardiac cells.

### Bmp signaling activity is asymmetrically increased on the left of the WT cardiac cone

**Figure 3:**
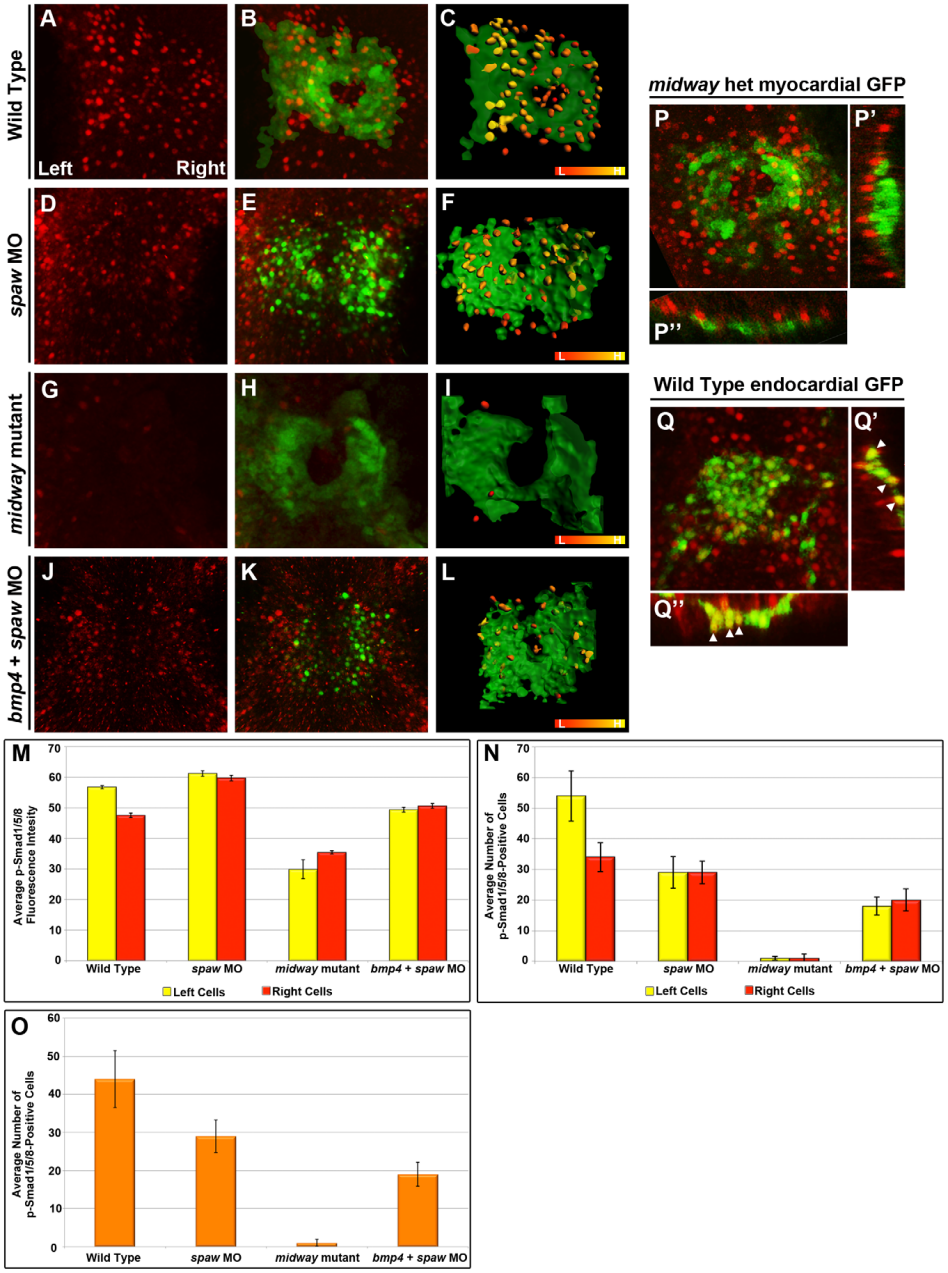
Quantitation of Bmp pathway activity by average fluorescence intensity and number of p-Smad1/5/8 positive cells. Immunofluorescence images for activated Smad1/5/8 (A,B,D,E,G,H,J,K,P–P″) in *Tg(myl7:eGFP)* embryos (myocardial GFP in B,E,H,K,P–P′″) or in *Tg*(*kdrl*:*egfp*) embryos (endocardial GFP in Q–Q″). C,F,I,L: Schematics of B, E, H and I using IMARIS surface tool. GFP signal in green; p-Smad1/5/8 positive cells are labeled according to intensity using the red (low) to yellow (high) spectrum ranging from an intensity value of 35–65, respectively. All images are dorsal views except for optical cross sections generated in IMARIS along the anterior/posterior axis (P′, Q′) or across the myocardium (P″, Q″). A–C: p-Smad1/5/8 fluorescence intensity is higher on the left of the WT cardiac cone (n = 6). D–F: p-Smad1/5/8 fluorescence intensity on both sides of the cone in *spaw* morphants is similar to that observed in cells on the left in WT (n = 6). G–I: *midway* mutants exhibit reduced fluorescence intensities and numbers of p-Smad1/5/8 positive cells, while p-Smad levels elsewhere in the embryo are seemingly unaffected (data not shown, n = 3). J–L: *bmp4* and *spaw* double morphants exhibit diminished p-Smad1/5/8 compared with WT (n = 6) but not as severe as observed in *midway* mutants. M: Comparison of the average fluorescence intensities of p-Smad1/5/8 positive cells on the left and right of the cardiac cone. N: Comparison of the average number of p-Smad1/5/8 positive cells on the left and right of the cardiac cone. O: Comparison of the average number of p-Smad1/5/8 positive cells in the entire cardiac field. P–P″: The myocardium shows little to no overlap of p-Smad1/5/8 positive cells (red) with the green of the myocardium. Q–Q″: Conversely, p-Smad1/5/8 (red) is observed to colocalize with the GFP positive endothelial cells in *Tg*(*kdrl*:*egfp*), indicated with arrowheads in Q′ and Q″, indicating that Bmp signals more strongly to the endocardium. Error bars indicate standard error of the mean. L: Low. H: High. MO: Morpholino. Het: heterozygote.

The negative influence of Bmp signaling on cell migration rate, along with the significantly slower velocities of right-sided cardiac cells in WT embryos, suggests that the Bmp pathway normally acts on the right of the cone to influence cardiac laterality. However, previous work has established that *bmp4* is expressed with a *left* bias at 20 hpf and that the laterality of this expression is altered in embryos with defects in jogging asymmetry [13]. Given the inconsistencies between earlier reports and our cell migration data, we were interested in determining the specific localization and potential asymmetry of Bmp activity within the heart. To this end, we analyzed Bmp pathway activation in WT embryos by immunofluorescence for the activated Bmp intracellular effectors Smads 1, 5 and 8 (phospho-Smad1/5/8 or p-Smad1/5/8). Consistent with previous reports [12], we find that Bmp pathway activity is asymmetrically increased on the left of the cardiac cone at 20 hpf, as indicated both by increased p-Smad1/5/8 fluorescence intensity in cells on the left compared with right (p<10^−3^) and by greater numbers of p-Smad1/5/8 positive cells present on the left of the cardiac cone (p = 0.003; Figure 3A–3C, 3M–3O).

At first glance, this left-biased increase in pSmad1/5/8 in cells with faster velocities appears contradictory to data from our time lapse experiments which strongly support a role for the Bmp pathway in negatively regulating myocardial migration rates. Results from our time-lapse analyses, coupled with our genetic data demonstrating BMP signaling is dispensable for generating asymmetry in the heart in the presence of asymmetric Nodal signaling, suggest that regardless of the increase in p-Smad1/5/8 on the left, the cue from Nodal for cells to increase migration rates is stronger than the influence of Bmp signals on these same cells. By contrast, as cells on the right of the cone do not receive inductive cues from Spaw, Bmp activation on the right significantly diminishes migration rates. In WT embryos, this leads to cell velocities on the right of the cone being reduced compared to those on the left. Thus, when Nodal signaling is absent (as in *spaw* morphants and SB-505124-treated embryos), all cells in the cone respond to repressive cues from the Bmp pathway and both left and right myocardial cell velocities are substantially reduced. Likewise, when the Nodal pathway is activated on both sides of the cone (as in *ntl* morphants) cell velocities are increased to higher rates than those observed in WT cells exposed to Nodal, presumably because bilateral Nodal signaling diminishes the repressive effects of Bmp on myocardial migration rates.

### Spaw negatively regulates Bmp responsiveness on the left of the cardiac cone

While we observe an increase in p-Smad1/5/8 on the left side of the cardiac cone, we argue that Nodal signaling increases migration on the left and overrides repressive cues from Bmp. Consistent with this hypothesis, analysis of Bmp activity in the hearts of *spaw* morphants reveals a significant increase in the fluorescence intensity of p-Smad1/5/8 immunostaining in both right (p<10^−3^) and left (p = 0.002) cardiac fields compared with WT (Figure 3D–3F, 3M–3O). These results indicate that Nodal signaling limits the level of Bmp pathway activity, potentially by competing for the common intracellular effector Smad4, a mechanism of Nodal/Bmp antagonism that is known to occur during earlier stages of L/R patterning in zebrafish and other species [20], [21]. Interestingly, despite having higher intensities of p-Smad1/5/8 fluorescence, we find that the average number of p-Smad1/5/8 positive cells is significantly diminished in *spaw* morphants compared to WT (p = 0.02) (Figure 3O), which may indicate a role for Nodal signaling in both positive and negative regulation of Bmp pathway activation within the cardiac cone. These results suggest that Nodal signaling ensures the establishment of differential migration rates along the cardiac L/R axis in two ways; first, by directly increasing cell velocities on the left and second, by limiting the level of Bmp activity on the left. Ultimately, robust development of jogging asymmetry appears to require left-restricted activation of the Nodal pathway to increase migration rates and response to the Bmp pathway on the right of the cone, where Bmps serve to diminish migration velocities.

### Bmp activation occurs in endocardial cells in the heart

In our immunofluorescence experiments, we noticed that the GFP staining in myocardial cells did not significantly colocalize with the p-Smad1/5/8 present in the heart field (Figure 3P–3P′″). As endocardial cells are also localized to the midline by this stage of development, we hypothesized that Bmps signal more predominantly to the endocardium at 20 hpf. To address this possibility, we performed p-Smad1/5/8 staining in embryos with GFP expressed from the *kdrl* promoter, which labels endothelial and endocardial cells [22]. We observed significant colocalization of GFP and p-Smad1/5/8 in these embryos, indicating that Bmp activity is primarily upregulated within endocardial cells (Figure 3Q–3Q′″). By contrast, all direct Nodal targets that have been identified in the heart are expressed within the myocardial population [9], [12], [23], [24], [25]. Thus, the Nodal and Bmp pathways appear to act in parallel to establish asymmetries in cell migration velocities within the cardiac cone: Nodal pathway activation in the myocardium on the left leads to increases in migration rates while Bmp signaling in the endocardium limits myocardial migration, primarily on the right. Precedence for cross-regulation between the endocardium and myocardium during the earlier migration events leading to cone formation have been described [26], [27]. Thus, interplay between these two tissues is critical for at least two migrations during cardiac development.

### FoxH1 is required for responsiveness of cardiac cells to both Nodal and Bmp signals

While loss of the ligand Spaw or the co-receptor Oep results in randomized jogging (Figure 2E and 2F), embryos with a nonsense mutation in the Nodal transcription factor FoxH1 [28] display 78% midline hearts (Figure 2K). These results suggest that FoxH1 performs both Nodal-dependent and independent functions within the heart. Interestingly, *midway* jogging defects are strikingly similar to those of embryos lacking both Nodal and Bmp signaling (Figure 2H, 2J and 2K) suggesting that FoxH1 may be required for cardiac cell responsiveness to both TGFβ pathways. To address this possibility, we analyzed cardiac cell migration in *midway/foxH1* mutants. Cells on the left and right of the cardiac cone in *midway* mutants migrate with average velocities of 7.8 nm/s and 7.1 nm/s, respectively (Figure 1G and 1H; Video S6). These migration rates are significantly faster than those of cardiac cells in embryos lacking Spaw (p<10^−3^) or treated with the Nodal-inhibitor drug SB-505124 (p<10^−3^), confirming a Nodal-independent function for FoxH1 in establishing cardiac laterality (Figure 1C, 1D and 1H). Importantly, *midway* cardiac velocities are not statistically different than those of *bmp4/spaw* double morphants (p = 0.580), suggesting that FoxH1 is necessary for cardiac cells to respond to both Nodal and Bmp signals. Consistent with this idea, we observe a significant, bilateral decrease in p-Smad1/5/8 in the hearts of *midway* mutants compared with WT, both in fluorescence intensity (p<10^−3^) and in the number of p-Smad1/5/8 positive cells (p<10^−3^) (Figure 3G–3I, 3M–3O). Interestingly, *midway* mutants exhibit a more profound decrease in Bmp pathway activation than that observed in *bmp4/spaw* double morphants in both fluorescence intensity and number of p-Smad1/5/8 positive cells (p<10^−3^), indicating that loss of FoxH1 activity profoundly blocks cardiac cell responsiveness to Bmp signals (Figure 3J–3L, 3M–3O).

### A model for the generation of asymmetry in the zebrafish heart

**Figure 4:**
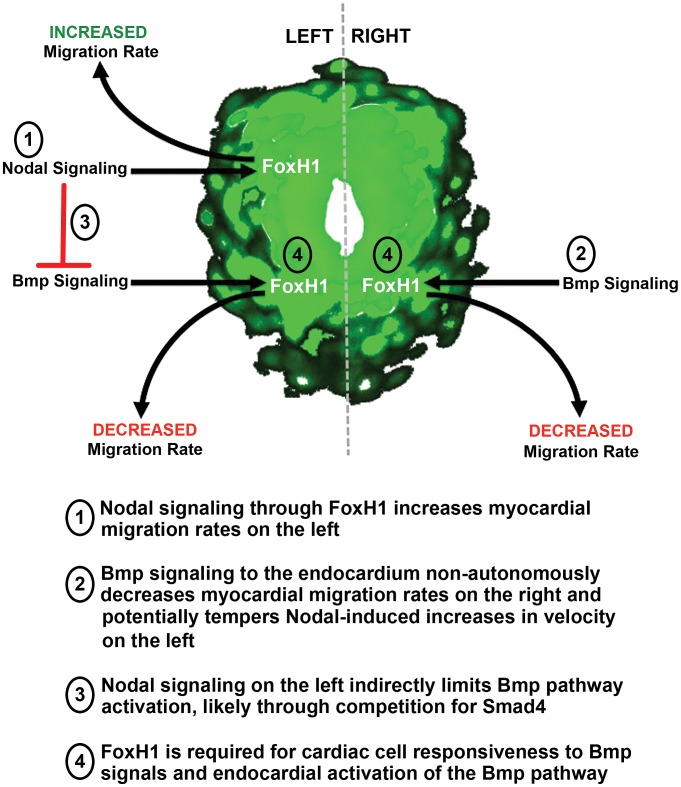
Model of the opposing roles for Nodal and Bmp signaling during asymmetric cardiac morphogenesis. 1: Nodal signaling to the myocardium through the transcription factor FoxH1 is required to asymmetrically increase cardiac migration rates specifically on the left of the cardiac cone. 2: The Bmp pathway is bilaterally activated in the endocardium but only effectively limits migration rates in the absence of strong inductive cues from Nodal signaling on the right of the cardiac cone. This leads to a non-cell autonomous decrease in myocardial migration rates on the right of the cone. 3: The Nodal pathway generally limits Bmp pathway activation in the heart, potentially through competition for access to proteins common to both pathways. 4: FoxH1 is also required in a Nodal-independent manner in the heart to mediate cardiac cell responsiveness to Bmp signals.

Here, we report the parallel requirements for Nodal and Bmp pathways in establishing cardiac laterality through opposing influences on cardiac cell migration rates (Figure 4). Additionally, we have uncovered a novel, Nodal-independent role for FoxH1 during this process in mediating cardiac cell responsiveness to Bmp signals (Figure 4). The Nodal-dependent activity of FoxH1 in regulating cardiac cell migration is likely restricted to myocardial cells, as the Nodal targets *lefty1, lefty2* and *has2* all display myocardial expression [9], [12], [23], [24], [25], the latter of which has been implicated in previous work [12] to influence myocardial migration. However, it is unclear at present whether the Nodal-independent function of FoxH1 is carried out within the myocardial or endocardial cells of the developing heart. FoxH1 can act as both transcriptional activator and repressor, and an inhibitory requirement for FoxH1 has been reported in the vasculature to limit expression of the VegF receptor, *kdrl* [29]. Consequently, FoxH1 may be required directly in the endocardium to block expression of Bmp antagonists or activate transcription of required components of the Bmp pathway. Alternatively, the influence of FoxH1 on Bmp activation may be more indirect. Recent work has reported that retinoic acid (RA) signaling is necessary for consistent asymmetry in *bmp4* expression in the zebrafish cardiac cone, with inhibition of RA leading to bilateral *bmp4* expression and an increase in midline jogging [30]. Interestingly, a genome-wide screen for FoxH1 binding sites revealed *aldh1a1*, a gene necessary for RA production, as a direct target of FoxH1 in mouse [31]. While zebrafish lack an ortholog of this specific RA processing enzyme, there are a number of other aldh1a family members expressed throughout zebrafish development [32], making it compelling to speculate that the loss of Bmp responsiveness in *midway* cardiac cells may be due to defects in RA signaling.

Our results support a model in which cardiac laterality is regulated by interactions and cross-regulations both between TGFβ pathways and between the myocardial and endocardial layers of the developing heart that regulate differential motility along the L/R axis. These interactions involve complex integrations between Nodal and Bmp pathways, and we demonstrate that cardiac cells are highly sensitive to the dosage of these TGFβ signals. Bilateral exposure to Spaw increases migration rates beyond what is observed in left cells of the WT cone, and loss of a single copy of *bmp4* in addition to Nodal signaling significantly alters both jogging laterality and cardiac cell velocities. Moreover, the signals that can influence laterality in the heart likely involve additional members of the TGFβ family. We note that inhibition of Nodal signaling with the SB-505124 drug decreases cell velocities as expected. However, jogging laterality in these embryos is predominantly midline, which differs from loss of Spaw or Oep (Figure 2E–2G). While this phenotype resembles that of embryos lacking Spaw and Bmp4 (Figure 2H and 2J), the cell migration rates in drug-treated embryos are consistent with loss of Nodal, but not Bmp signaling (Figure 1H) and, indeed, we find that the Bmp pathway is still activated within the heart field upon SB-505124 treatment (data not shown). The *spaw* morpholino completely abolishes expression of *spaw* in the LPM, strongly suggesting that Spaw is absent in the hearts of these embryos. This, coupled with the similar phenotypes of *spaw* knockdown and LZ*oep* mutants, suggests that the effect of the drug is not a result of more complete knockdown of Nodal signaling. SB-505124 acts intracellularly on the Alk 4/5/7 Type I receptors, which are utilized by both Nodal and TGFβ ligands. Overall, this suggests that another TGFβ molecule signaling through the Nodal receptors can affect the migration of cardiac cells and may be important for allowing the cardiac cells to respond to fluctuations in Bmp levels when Spaw is absent. Taken together, these results have implications for determining the underlying genetic lesions in CHD, as they suggest that heterozygous mutations in components of different TGFβ signaling pathways may synergize to produce severe phenotypes. Further analysis of integrations of signals within and between cardiac cells will provide insight into the general mechanisms driving asymmetric morphogenesis and will greatly enhance our understanding of the potentially complicated genetic interactions underlying the development of CHD in humans.

## Materials and Methods

### Zebrafish strains and genotyping

All WT and GFP transgenic fish pairs used to generate embryos for this analysis have been determined to exhibit low backgrounds of L/R phenotypes. The WT strains used in these experiments include Tu, Alb/+, WIK, and AB. The following additional strains were used in this report: *Tg(myl7:egfp)* [33], *bmp4^Y180^* mutants [20], *Tg(kdrl:egfp)* [22], LZ*oep^tz257^* mutants [18]. *MZbmp4^Y180^* mutants were generated as previously described [20]. Genotyping to identify embryos carrying the *bmp4^Y180^* and *midway* mutations was conducted as previously described [20], [28].

### Morpholino injections and SB-505124 drug treatment

Morpholinos were diluted in phenol red and 500 pL were injected into zebrafish embryos between the 1 and 4 cell stages. Both the antisense start-site *spaw* morpholino (5-GCACGCTATGACCGGCTGCATTGCG-3) [34] and the antisense start-site *ntl* morpholino (5-GACTTGAGGCAGGCATATTTCCGAT-3) [35] were injected at a 1 ng/500 pL concentration and the antisense splice-site *bmp4* morpholino (5-GGTGTTTGATTGTCTGACCTTCATG-3) [14] was injected at a 2 ng/500 pL concentration. The SB-505124 Nodal inhibitor drug [16] was reconstituted in DMSO and stored at 4°C at a concentration of 10 mmol. A 40 µM dilution of SB-505124 in blue water was added to embryos at tail bud stage.

### Whole-mount immunofluorescence

Embryos were fixed in 4% PFA at 4°C overnight followed by gradual rehydration into PBDT (1×PBS, 0.1% Tween 20, 1% DMSO). After removal of the tails, embryos were incubated in 10 µg/mL proteinase K for 10 minutes. Proteinase K activity was stopped with a 20 minute incubation in 4% PFA. Embryos were then blocked for 2 hours in PBDT containing 10% normal goat serum (NGS) and incubated overnight at 4°C in a 1∶100 dilution of monoclonal GFP antibody (Roche #11814460001) and 1∶100 dilution of p-Smad1/5/8 antibody (Cell Signaling Technology #9511) in PBDT. The following day, embryos were washed in PBDT and incubated at 4°C overnight in 1∶100 dilutions of CY3 donkey anti-rabbit and CY2 donkey anti-mouse. (Southern Biotech #1090-02). After washing, the embryos were imaged on a Leica SP5 spectral confocal microscope.

### Time-lapse imaging and analysis

Time-lapse imaging was performed as previous described [9]. Briefly, embryos were screened for GFP expression at the 18–19 somite stage and mounted in 2% low-melt agarose on the converslip bottom of a round dish with the cardiac field positioned directly adjacent to the coverslip. The agarose was covered with a solution of water and tricaine to immobilize the embryos throughout the course of the time lapse. Embryos were then imaged an average of 4.5 hours on an inverted Leica SP5 spectral confocal microscope using a heated stage set to 29°C. Multiple embryos (up to 6) were imaged during a single time lapse using the mark and find feature of the Leica software. Once collected, time lapse series were transferred to the Volocity software for analysis where each series was merged to a single plane at each time point. Velocity measurements reported were determined by Volocity software (Perkin-Elmer, USA).

Atrial cells were tracked for analysis as these are the cells that we and others report to exhibit the most pronounced asymmetric migrations that drive the process of cardiac jogging [9], [10], [11], [12]. In all embryos analyzed, cells were defined as being “left’ or “right” due to their position along the L/R axis of the cardiac cone. In embryos that lack asymmetry in cell migration within the cone (all genotypes analyzed other than WT), left and right cells migrate along the lateral edges of the cone. However, cells at the posterior of the cone migrated directly towards the anterior with straight trajectories and not along the lateral edges. Given the different migration phenotype of these cells compared to cells from the left or right, we designated them as “center” in our analysis. The “center” cells in these embryos are those that ultimately involute during formation of the linear heart tube. Thus, to keep the “center” designation consistent between WT and other genotypes, we label the cells that normally involute in WT [11] as “center” cells in these embryos. Cells labeled as “left” in wildtype are within the *lefty2* expression domain [9] indicating that these cells are exposed to Spaw signals while cells labeled as “right” in wildtype are those lacking expression of Spaw downstream targets and are thus not exposed to Nodal signaling.

### p-Smad1/5/8 image processing

All images of p-Smad1/5/8 immunofluorescence were taken on an SP5 spectral confocal microscope and all images were taken at the same laser intensity and gain settings. To ensure that no significant variations in fluorescence intensity were detected due to artifact, a minimum of three separate immunostaining experiments were performed and analyzed for embryos of a single genotype, with these embryos being imaged on separate days. A minimum of three embryos were analyzed for p-Smad1/5/8 fluorescence for each genotype. We normalized all images to the right cells in the wildtype controls and each of the three different trials in wildtype embryos produced equivalent results. Image analysis was performed using the IMARIS software (Bitplane Software, USA). The green surface of the myocardium was generated using the surface tool, and the region of interest for identification of p-Smad1/5/8 positive cells was determined to be the GFP positive portion of the image. The p-Smad1/5/8 positive nuclei were selected using the surface tool, allowing spot separation. The field of each image was then split into left and right sides.

### Statistical analysis

Student T-tests were used to make all statistical comparisons. In order to test the null hypothesis that the triplet distributions of left, center, and right cell velocities were identical for data collected from two different genotypes, data were normalized as follows. For each of the three spatial designations, the mean and standard deviation were calculated for all data pooled across both genotypes. The mean was subtracted from the pooled data, and the resulting values were divided by the standard deviation. Normalized data were then re-pooled across all three designations separately for both genotypes, and the comparison between genotypes was performed. Comparisons between left cell velocities of one genotype and left cell velocities of another were made directly, without normalization. In each analysis, p values less than 0.05 were considered to be statistically significant.

## Supporting Information

Video S1 Myocardial Migration in a WT embryo. Time lapse movie of a *Tg(myl17:eGFP)* embryo beginning at 19–20 hpf. Myocardial cells on the left of the cone migrate toward the anterior and left significantly faster than do cells on the right. This results in displacement of the entire cone toward the left and extension of the forming linear heart tube along the anterior-posterior (A/P) axis in a leftward direction.
(AVI)

Video S2 Myocardial Migration in a *ntl* morphant. Time lapse movie of a *Tg(myl17:eGFP)* embryo injected with *ntl* morpholino beginning at 19–20 hpf. There is no obvious asymmetry in myocardial migration rate along the L/R axis, with all cells migrating faster than cells of the left myocardium in WT. This results in the cardiac cells of *ntl* morphants displaying trajectories directed toward the anterior with no clear L/R displacement. This results in a rapid movement of the entire cone toward the anterior of the embryo, with slightly precocious extension of the linear heart tube along the A/P axis.
(AVI)

Video S3 Myocardial Migration in a *spaw* morphant. Time lapse movie of a *Tg(myl17:eGFP)* embryo injected with *spaw* morpholino beginning at 19–20 hpf. There is no obvious asymmetry in myocardial migration rate along the L/R axis and all cells have trajectories directed toward the anterior with no clear L/R displacement. This results in a slow movement of the entire cone toward the anterior of the embryo, with extension of the linear heart tube along the A/P axis significantly delayed and not visible during the course of this time lapse.
(AVI)

Video S4 Myocardial Migration in a *bmp4^Y180^* heterozygote injected with *spaw* morpholino. Time lapse movie of a *Tg(myl17:eGFP)/bmp4^Y180^* ^+/−^ embryo injected with *spaw* morpholino beginning at 19–20 hpf. There is no obvious asymmetry in myocardial migration rate along the L/R axis and all cells have trajectories directed toward the anterior with no clear L/R displacement. However, cell velocities are significantly faster than those of *spaw* morphants.
(AVI)

Video S5 Myocardial Migration in a *bmp4/spaw* double morphant. Time lapse movie of a *Tg(myl17:eGFP)* embryo injected with both *bmp4* and *spaw* morpholinos beginning at 19–20 hpf. There is no obvious asymmetry in myocardial migration rate along the L/R axis and all cells have trajectories directed toward the anterior with no clear L/R displacement. However, cell velocities are significantly faster than those of *spaw* morphants.
(AVI)

Video S6 Myocardial Migration in a *midway* mutant. Time lapse movie of a *Tg(myl17:eGFP)/midway* mutant embryo beginning at 19–20 hpf. There is no obvious asymmetry in myocardial migration rate along the L/R axis and all cells have trajectories directed toward the anterior with no clear L/R displacement. The cell velocities are significantly faster than those of *spaw* morphants and are not statistically different than those of *bmp4/spaw* double morphants.
(AVI)
